# Evaluation of methicillin-resistant *Staphylococcus aureus* (MRSA) bacteremia: Epidemiology, clinical characteristics, and outcomes in the older patients in a tertiary teaching hospital in Malaysia

**DOI:** 10.1186/s12879-023-08206-y

**Published:** 2023-04-18

**Authors:** Kejal Hasmukharay, Soo Tein Ngoi, Nor Izzati Saedon, Kit Mun Tan, Hui Min Khor, Ai Vyrn Chin, Maw Pin Tan, Adeeba Kamarulzaman, Nuryana binti Idris, Wen Kiong Niek, Cindy Shuan Ju Teh, Shahrul Bahyah binti Kamaruzzaman, Sasheela Sri La Sri Ponnampalavanar

**Affiliations:** 1 Geriatric Unit, Department of Medicine, Faculty of Medicine, Universiti Malaya, Kuala Lumpur, 50603 Malaysia; 2 Department of Anaesthesiology, Faculty of Medicine, Universiti Malaya, Kuala Lumpur, 50603 Malaysia; 3 Infectious Disease Unit, Department of Medicine, Faculty of Medicine, Universiti Malaya, Kuala Lumpur, 50603 Malaysia; 4 Department of Medical Microbiology, Faculty of Medicine, Universiti Malaya, Kuala Lumpur, 50603 Malaysia

**Keywords:** Healthcare-associated, Hospital-acquired, Hypoalbuminemia, Methicillin-resistant *Staphylococcus aureus* (MRSA), Older adults, Risk factors

## Abstract

**Background:**

Methicillin-resistant *Staphylococcus aureus* (MRSA) bacteremia is a major concern in the global healthcare system. However, data from Asian regions dealing with the singularity of this infection in older persons is lacking. We aimed to identify the differences in the clinical characteristics and outcomes of MRSA bacteremia between adults aged 18–64 and ≥ 65 years.

**Methods:**

A retrospective study cohort was conducted at the University Malaya Medical Centre (UMMC) on cases of MRSA bacteremia from 2012 to 2016. Patient demographic and clinical data were collected for risk factors analyses.

**Results:**

New cases of MRSA bacteremia showed a trend of increase from 0.12 to 100 admissions in 2012 to 0.17 per 100 admissions in 2016 but a drop was observed in 2014 (0.07 per 100 admissions). Out of the 275 patients with MRSA bacteremia, 139 (50.5%) patients were aged ≥ 65 years old. Co-morbidities and severity at presentation were significantly higher among older adults, including diabetes mellitus (p = 0.035), hypertension (p = 0.001), and ischemic heart disease (p < 0.001), as well as higher Charlson Comorbidity Index (p < 0.001) and Pitt bacteremia scores (p = 0.016). Central line-associated bloodstream infections were more common among younger patients (37.5% vs. 17.3% in older patients, p < 0.001), while skin and soft tissue infections are more frequent among older adults (20.9% vs. 10.3% in younger patients, p = 0.016). All-cause mortality and in-hospital mortality were significantly higher in older patients (82.7% and 56.1% vs. 63.2% and 28.7% in younger patients, p < 0.001). Multivariate analysis revealed age ≥ 65 years (adjusted odds ratio: 3.36; 95% confidence interval: 1.24–9.13), Pitt score ≥ 3 (2.15; 1.54–3.01), hospital (6.12; 1.81–20.72) and healthcare (3.19; 1.30–7.81) acquisition of MRSA, indwelling urinary catheters (5.43; 1.39–21.23), inappropriate targeted treatment (8.08; 1.15–56.86), lack of infectious disease team consultation (2.90; 1.04–8.11) and hypoalbuminemia (3.31; 1.25–8.79), were significant risk factors for 30-day mortality.

**Conclusion:**

Older patients’ risk of mortality from MRSA bacteremia was three times higher than younger patients. Our data will contribute to developing and validating a robust scoring system for risk-stratifying patients to achieve better management and improved clinical outcomes.

## Background

The global population aged 65 years and above has increased from 7.6 to 9.3% within the past decade [1]. By 2050, 65 years and older individuals are estimated to represent 15.9% of the global population [1]. Currently, Malaysia is officially considered an aging nation since the population aged 65 years and above has reached 7.3% of the total population in 2022 [2]. Increasing age is associated with increased susceptibility to ill health, including infections, which will have a great impact on medical and socioeconomic systems in a developing country. Co-morbid illnesses, exposure to medical devices, multiple procedures, institutionalization, frailty, sarcopenia, malnutrition, and disability may all contribute to higher rates of infection in older patients. In general, infections are more frequent in older individuals compared to young adults and carry higher risks of hospitalization and mortality [3]. Despite antimicrobial therapy, older patients experience poorer outcomes due to delayed therapy and diagnosis because of vague and atypical disease presentations [4].

*Staphylococcus aureus* is a Gram-positive bacterium that colonizes the nares, axillae, vagina, pharynx, and damaged skin surfaces. *S. aureus* has been identified as an important etiologic agent of nosocomial infections [5]. While *S. aureus* usually leads to just minor skin infections (e.g., folliculitis) [6], penetration into deeper tissues and the bloodstream through the injured skin or mucosal membranes could result in severe infections, leading to bacteremia [7]. Even with surgical procedures and antimicrobials, bacteremia caused by *S. aureus* is often associated with high morbidity and mortality [7].

*S. aureus* is essentially classified into two groups based on antibiotic susceptibility: methicillin-susceptible *S. aureus* (MSSA) and methicillin-resistant *S. aureus* (MRSA) [8]. MRSA infections are reportedly associated with a significant increase in all-cause mortality, infection-attributable mortality, intensive care unit (ICU) mortality, post-infection length of stay, length of stay in ICU, and septic shock [9]. In 2019, over 100,000 deaths have been attributed to antimicrobial resistance worldwide, with MRSA accounting for a large proportion of these deaths [10]. Asia represents a region with the highest prevalence of MRSA, with reports in Southeast Asian countries suggesting a highly variable prevalence of 2–80% [9, 11]. When it was first discovered, MRSA was considered a nosocomial infection. However, since the late 1980s, the emergence and worldwide dissemination of community-acquired MRSA have been documented [8, 12]. MRSA is the major cause of global *S. aureus* bacteremia cases and is often associated with higher mortality rates and poorer clinical outcomes compared to MSSA [13]. Given the increasing level of threats and changing epidemiology, timely updates of the surveillance on the epidemiology, clinical characteristics, and outcomes of MRSA bacteremia are essential.

This study aims to determine the differences in clinical characteristics and outcomes of MRSA bacteremia between patients aged 18–64 years and those aged 65 years and over and to identify factors associated with increased risk of infection. The findings of this study will inform future epidemiological, clinical and molecular studies to guide the development of evidence-based management of MRSA bacteremia.

## Materials and methods

### Study population and clinical data collection

This retrospective observational study cohort was conducted at the University Malaya Medical Centre (UMMC), a tertiary teaching hospital located in Kuala Lumpur, Malaysia. Ethics approval was obtained from the UMMC medical research ethics committee (MEC-ID: 20145-168). Consecutive patients who had MRSA isolated from blood cultures taken between 1st January 2012 to 31st December 2016 were identified from the Medical Microbiology Diagnostic Laboratory database. All adult patients (aged 18 years and older) with MRSA bacteremia during this period were included. A standardized case report form was used to collect all required data extracted from patient case notes (for cases between January 2012 – September 2014) and the hospital electronic medical records (for cases from October 2014 onwards). Demographic and clinical data collected included age, sex, ethnic origin, co-morbidities, length of hospital stay, source of acquisition (hospital, healthcare-associated or community), site of infection, previous antibiotic use, principal clinical manifestations, presence of indwelling devices, source control, antibiotic treatment, follow-up, and outcomes at 14 days, 30 days, 90 days, and 6 months after first positive culture, relapses, and recurrences of MRSA bacteremia.

### Incidence, comorbidity, and severity of bacteremia

The MRSA bacteremia cases/100 admissions were calculated using the total adult admissions for the respective years as the denominator. Co-morbidities and severity of sepsis were measured by the Charlson Comorbidity Index [14] and Pitt bacteremia scores [15], respectively.

### Sources of methicillin-resistant *Staphylococcus aureus* acquisition

Individuals who had MRSA identified from at least one blood culture with or without clinical signs and symptoms of infection were identified. The sources of MRSA acquisition were categorized according to the Friedman criteria [16]: hospital-acquired MRSA (HA-MRSA), healthcare-associated (HCA-MRSA), or community-acquired (CA-MRSA). HA-MRSA was defined as isolation of MRSA from blood cultures taken greater than 48 h after admission but there were no signs or symptoms of infection at admission. HCA-MRSA was defined as bacteremia in patients that were admitted to the healthcare system within the past 3 months or within 30 days post-operation, if the MRSA was isolated from a surgical site infection, or within a year of operation for medical device implant. These included patients from a nursing home or were on hemodialysis. Isolation of MRSA from the blood within 48 h of hospital admission with the risk factors mentioned above was labeled as HCA-MRSA. CA-MRSA was defined as isolation of MRSA from the blood samples obtained within 48 h of admission and the episode did not fit the above conditions of HCA-MRSA. The classification of bloodstream infections into HA, HCA, and CA sources is supported by the epidemiologic, microbiologic, and outcome characteristics of these three categories of infections [17, 18].

### Sources of bacteremia

The bacteremia sources were defined according to the Centre for Disease Control and Prevention (CDC) criteria [19]. Briefly, central line-associated bloodstream infections (CLABSI) were confirmed by the recovery of *S. aureus* from two or more blood cultures in a patient who had a central line at the time of infection or within 48 h before the development of infection. Surgical site infection (SSI) was defined based on the CDC criteria [19]. Catheter-associated urinary tract infections (CA-UTI) were considered symptomatic urinary tract infections in a patient who had an indwelling urinary catheter at the time of or within 48 h before infection onset. Primary bacteremia was defined as bacteremia which occurred in the absence of an apparent portal of entry and other sites of infection after careful examination of the clinical, laboratory, and imaging data. Skin and soft tissue infection (SSTI) was defined according to the Infectious Diseases Society of America (IDSA) guidelines [20].

### Management

Empirical antibiotic use was affirmed if administered after the onset of symptoms but before microbiological information was obtained. Initial antibiotic treatment was considered appropriate if the strain was susceptible to at least one of the antibiotics administered according to the Clinical and Laboratory Standards Institute (CLSI) breakpoints during the time of analysis except for aminoglycosides, which were considered inappropriate regardless of the susceptibility test results [21].

The management strategies employed include the request for a transthoracic or transesophageal echocardiogram (if clinically indicated based on guideline recommendations), the number of repeat cultures obtained every 72 h until culture negative, removal of implanted prostheses and/or catheters, initiation of an antimicrobial agent with activity against MRSA and duration of treatment was recorded [22]. Definitive antimicrobial referred to the administration of antibiotics after the availability of culture and sensitivity results. Antibiotic treatment was considered appropriate if the strain was susceptible to at least one of the administered antibiotics including vancomycin, daptomycin, linezolid, tigecycline, and ceftaroline. If the primary team requires further advice in the management of a patient with bacteremia, an infectious disease (ID) consultant is sought, who will then review and advise on the management as per the hospital’s guidelines.

### Outcomes

The outcomes recorded include persistent and recurrent bacteremia, mortality, and length of stay. Persistent was defined as bacteremia of 7 days or longer despite optimal treatment [23]. Recurrent bacteremia was defined as the recurrence of the bacteremia occurring 14 days or more after blood cultures had turned negative [24]. All-cause mortality at days 14, 30, and 6 months from the first positive blood culture was documented. The follow-up on patient mortality after discharge was done by contacting the National Registration Department to obtain data on deaths for all patients included in this study. The follow-up period was up to 180 days from the end of the study in December 2016. Readmission data was only collected for patients who had been re-admitted to UMMC. The length of stay was defined as the termination of stay (discharge alive/dead) after the onset of bloodstream infection [25].

### Statistical analysis

All statistical analyses were performed using SPSS version 25 (SPSS Inc., Chicago, IL, USA). Baseline data including patients’ demography, clinical characteristics, disease severity, and clinical and treatment outcomes were compared between patients aged 18 to 64 years and those aged 65 years and above. Continuous variables were represented as mean ± standard deviation (SD) and compared using the Student t-test. Categorical variables were represented as count (n) and frequency (%) and compared using the Chi-squared or Fisher’s exact test, as appropriate. Statistical significance was established at p < 0.05. All variables were included in a multiple regression analysis to determine the significant predictors of early (14-day) and 30-day mortality. The risk factors, or factors associated with mortality, were expressed as adjusted odds ratios (AOR) with 95% confidence intervals (CI). Survival probabilities at 14, 30, and 180 days were compared between the two age groups using the Log-rank test. A Kaplan-Meier survival curve was plotted to compare the cumulative survival of the two age groups at three and six months.

## Results

### Annual incidence rates of methicillin-resistant Staphylococcus aureus bacteremia

A total of 275 unique cases of MRSA bacteremia were identified between 2012 and 2016. Table 1 summarizes the annual number of cases per 100 admissions. The overall MRSA bacteremia rates ranged between 0.12 and 0.17 per 100 admissions, with a lower number of cases documented in the years 2014 and 2015. The cases of HA-MRSA infections per 100 admissions were generally higher than that of the HCA- and CA-MRSA infections, except for the year 2016 which documented a higher number of HCA-MRSA cases. The yearly incidence of MRSA bacteremia was higher in the older compared to the younger patient population. In 2012, the number of cases per 100 admissions in patients aged 65 years and older was 0.22 per 100 admissions compared to 0.08 per 100 admissions in adults aged less than 65 years and this rose to 0.33 per 100 admissions compared to 0.11 per 100 admissions respectively in 2016.

**Table 1:** Methicillin-resistant *S. aureus* bacteremia rates from 2012 to 2016

Year	No. of cases	Total adult admissions /year	Cases/100 admissions			
			**Overall**	**HA-MRSA**	**HCA-MRSA**	**CA-MRSA**
2012	54	44,836	0.12	0.08	0.02	0.02
2013	64	46,575	0.13	0.08	0.05	0.01
2014	36	49,681	0.07	0.04	0.02	0.01
2015	44	45,494	0.10	0.05	0.03	0.01
2016	77	44,675	0.17	0.07	0.09	0.02

MRSA: methicillin resistance staphylococcus aureus; HA: hospital-acquired; HCA: healthcare-associated; CA: community-acquired

### Demographics, clinical characteristics, and illness severity by age group

Approximately half of the patients (n = 139, 50.5%) were aged 65 years and older. Male patients (n = 165, 60%) were more represented than female patients (n = 110, 40%). Most of the patients were of Malay (n = 98, 35.6%) and Chinese (n = 97, 35.3%) ethnicities, followed by Indians (n = 76, 27.6%) and others (n = 4, 1.5%). The average length of hospital stay was 24.2 days with a 19.3% ICU admission rate. Most of the patients were previously hospitalized (88%) with the majority of them reporting a recent hospitalization (less than three months ago; 60.7%). More than half of the MRSA bacteremia cases were associated with HA infections (52.7%), followed by HCA (35.6%) and CA (11.6%) infections. Approximately 66.3% of the patients with HCA infection had been previously admitted to UMMC.

A comparison of patients’ demography and clinical characteristics between age groups revealed significant differences in ethnicity (p ≤ 0.001), more common use of continuous bladder drainage (p = 0.003), and other devices (chest tube, nasogastric (NG) tube, prosthetic valve, pacemaker, prosthetic implants, or cerebral shunts) (p = 0.016), and higher rates of diabetes mellitus (p = 0.035), hypertension (p = 0.001), and ischemic heart disease (p < 0.001) among the older patients (Table 2). Similarly, older patients had higher Charlson Comorbidity Index (p < 0.001) and Pitt bacteremia scores (p = 0.016) than patients aged < 65 years old. The rate of CLABSI was significantly higher in patients aged < 65 years old (p < 0.001) while skin and soft tissue infections were more common among older individuals (p = 0.016). A greater number of MRSA bacteremia in patients aged < 65 years old were associated with removable sources such as central lines (p = 0.005). A total of 76 patients had polymicrobial infections and the organisms identified were mainly methicillin-resistant coagulase-negative staphylococci (n = 36), *Enterococcus faecalis* (n = 12), pan-susceptible *Acinetobacter baumannii* (n = 8), pan-susceptible *Klebsiella pneumoniae* (n = 13), and *Enterococcus faecium* (n = 7).

**Table 2:** Clinical characteristics by age groups

	18–64 years (n = 136, 49.5%)	≥ 65 years (n = 139, 50.5%)	p-value
Age, mean (SD)^a^ (years)	49.34 (12.03)	74.94 (7.20)	< 0.001*
Gender, n (%)			
Male	84 (61.8)	81 (58.3)	0.555
Female	52 (38.2)	58 (41.7)	0.555
Ethnicity, n (%)			
Malay	47 (34.6)	51 (36.7)	0.712
Chinese	35 (25.7)	62 (44.6)	0.001*
Indian	50 (36.8)	26 (18.7)	< 0.001*
Others	4 (2.9)	0 (0)	0.058
Classification, n (%)			
Hospital-acquired	70 (51.5)	74 (53.2)	0.769
Healthcare-associated	53 (39.0)	46 (33.1)	0.310
Community-acquired	13 (9.6)	19 (13.7)	0.288
Length of stay in survivors, mean/n (%)	29.5/97 (71.3)	22.6/61 (43.9)	0.417
Intensive Care Unit admission, n (%)	24 (17.6)	29 (20.9)	0.499
Devices in situ during infection, n (%)			
Peripheral venous line	134 (98.5)	137 (98.6)	0.982
Central venous lines	36 (26.5)	25 (18.0)	0.090
Continuous bladder drainage	50 (36.8)	76 (54.7)	0.003*
Others (chest tube, nasogastric tube, prosthetic valve, pacemaker, prosthetic implants, or cerebral shunt)	17 (12.5)	33 (23.7)	0.016*
Previous hospitalization, n (%)			
Less than 3 months	89 (65.4)	78 (56.1)	0.168
3 months or more	28 (20.6)	47 (33.8)	0.092
None	19 (13.2)	14 (10.1)	0.238
Co-morbidities, n (%)			
Chronic Kidney Disease	71 (53.0)	86 (62.8)	0.103
End Stage Renal Failure	27 (20.1)	16 (11.7)	0.056
Diabetes Mellitus	70 (51.5)	89 (64.0)	0.035*
Hypertension	74 (54.4)	103 (74.1)	0.001*
Airway diseases	4 (3.0)	10 (7.2)	0.109
Ischemic Heart Disease	11 (8.1)	38 (27.3)	< 0.001*
Others	66 (48.5)	68 (48.9)	0.948
Infection-severity and biochemical data			
Pitt bacteremia score ≥ 3, n (%)	17 (12.5)	33 (23.7)	0.016*
Charlson score, mean (SD)	4.14 (2.83)	6.65 (3.05)	< 0.001*
Charlson score > 4, n (%)	59 (43.4)	111 (79.9)	< 0.001*
Albumin, mean (SD)	26	25	0.514
Hypoalbuminemia (albumin < 29 g/L), n (%)	90 (66.1)	99 (71.2)	0.367
Exposure to antibiotics in the last 90 days, n (%)	88 (64.7)	84 (60.4)	0.673
Types of antibiotics exposure in the last 90 days, n (%)			
Beta lactams	76 (86.4)	76 (90.5)	0.400
Glycopeptide (Vancomycin)	15 (17.0)	8 (9.5)	0.147
Fluoroquinolone (Ciprofloxacin)	11 (12.5)	10 (11.9)	0.905
Polymyxin (Colistin)	4 (4.5)	3 (3.6)	0.747
Macrolide	5 (5.7)	7 (8.3)	0.495
Lincosamide (Clindamycin)	1 (1.1)	0 (0)	0.327
Others	24 (27.3)	15 (17.9)	0.140
Site of Infection, n (%)			
Primary bacteremia	39 (28.7)	51 (36.7)	0.157
Central line-associated bloodstream infection	51 (37.5)	24 (17.3)	< 0.001*
Respiratory (community/hospital-acquired pneumonia and ventilator-associated pneumonia)	20 (14.7)	27 (19.4)	0.299
Skin and soft tissue infection	14 (10.3)	29 (20.9)	0.016*
Bones and joints	4 (2.9)	4 (2.9)	0.975
Others	8 (5.9)	4 (2.9)	0.223
Poly-microbial bacteremia, n (%)	37 (27.2)	39 (28.1)	0.796
Removable Sources, n (%)	71 (55.0)	55 (41.0)	0.020*
Pus / Collections	15 (21.1)	18 (32.7)	0.142
Line	52 (73.2)	27 (49.1)	0.005*
Others	4 (5.6)	10 (18.2)	0.026*

*p-value < 0.05 indicates statistical significance

^a^SD: Standard deviation

### Treatment, clinical outcomes, and mortality for patients by age group

Table 3 summarizes the treatment and clinical outcomes of MRSA bacteremia patients from both age groups. Most of the patients received empirical therapy (> 97% in both groups). However, only 14–18% of the empiric treatments were effective. Vancomycin (71–76%) was most frequently prescribed in targeted treatments, followed by a combination of vancomycin and linezolid (1–2%), and linezolid alone (≤ 1.5%). Approximately 20–26% of the patients did not receive targeted antibiotic treatment. Older patients had significantly shorter treatment duration than patients aged < 65 years old (p = 0.017), differing by 5 days on average. However, more than half of the older patients (56.6%) did not receive adequate treatment duration for MRSA bacteremia and only 52.5% of the older patients had repeated blood cultures. A significantly greater proportion of patients in the younger age group (< 65 years old) received appropriate treatment duration (p = 0.005) and had repeated blood cultures (p = 0.010). Generally, less than half of the patients in both age groups (30–41%) had obtained an expert infectious disease team consultation. A quarter of the patients had persistent bacteremia (25–27%) but recurrent bacteremia was less frequent (5–9%). All-cause mortality and in-hospital mortality were significantly higher among older patients (82.7% and 56.1%, respectively) compared to patients aged < 65 years old (63.2% and 28.7%, respectively) (p < 0.001). Similarly, the mortality at 14 (p = 0.002), 30, and 180 days (p < 0.001) were significantly higher among older patients. In our cohort, four patients had passed away within 48 h of blood cultures being taken, all of them were more than 65 years old, had HA bacteremia and one was on effective antibiotics at the time of death.

**Table 3:** Treatment and clinical outcomes of patients by age groups

	18–64 years (n = 136)	≥ 65 years (n = 139)	p-value
Empirical antibiotics, n (%)	133 (97.8)	137 (98.6)	0.760
Effective MRSA empiric therapy, n (%)	19 (14.2)	25 (18.3)	0.374
Targeted MRSA treatment, n (%)	108 (80.6)	102 (73.4)	0.515
Vancomycin	103 (75.7)	99 (71.2)	0.397
Linezolid	2 (1.5)	1 (0.7)	0.549
Vancomycin + Linezolid	3 (2.2)	2 (1.4)	0.303
None	28 (20.6)	37 (26.6)	0.239
Treatment duration, mean (SD)^a^ days	16.27 (18.1)	11.65 (13.4)	0.017*
Appropriate treatment duration, n (%)	102 (94.4)	46 (43.4)	0.005*
Infectious disease team consult, n (%)	45 (41.3)	32 (30.5)	0.100
Repeated blood cultures obtained, n (%)	92 (67.6)	73 (52.5)	0.010*
Persistent bacteremia, n (%)	37 (27.2)	35 (25.2)	0.281
Recurrent bacteremia, n (%)	12 (8.9)	8 (5.8)	0.422
Overall all-cause mortality, n (%)	86 (63.2)	115 (82.7)	< 0.001*
In hospital mortality, n (%)	39 (28.7)	78 (56.1)	< 0.001*
Early mortality (14 days), n (%)	21 (15.4)	44 (31.7)	0.002*
Mortality in 30 days, n (%)	32 (23.5)	67 (48.2)	< 0.001*
Mortality in 180 days, n (%)	61 (44.9)	100 (71.9)	< 0.001*

*p-value < 0.05 indicates statistical significance

^a^SD: Standard deviation

### Risk factors associated with mortality

All variables including patient demographic data, clinical characteristics, disease severity, treatment, and clinical outcomes were assessed to determine the risk factors for early (14-day) and 30-day mortality in the patients with MRSA bacteremia. Multivariate analysis revealed that old age (≥ 65 years old), infections caused by HCA-MRSA, hypoalbuminemia (< 29 g/L), and inappropriate targeted treatment for MRSA bacteremia were significantly associated with 30-day mortality (Table 4). On the other hand, high Pitt scores (> 3), the presence of an indwelling urinary catheter, lack of infectious disease team consultation, and HA-MRSA infections were presented as significant risks for both 14-day (early) and 30-day mortality. The presence of polymicrobial bacteremia was indicated as a significant risk factor for early mortality (AOR 3.35, 95% CI 0.43–1.07, p = 0.032).

**Table 4:** Risk factors associated with 14-day and 30-day mortality in patients with MRSA bacteremia

Risk factors	Adjusted Odds Ratio with 95% Confidence Interval			
	**14-day Mortality**	**p-value**	**30-day Mortality**	**p-value**
Age ≥ 65 years	3.19 (0.89–11.50)	0.311	3.36 (1.24–9.13)	0.024*
Pitt score ≥ 3	2.11 (1.52–2.93)	< 0.001*	2.15 (1.54–3.01)	< 0.001*
Charlson score > 4	1.06 (0.87–1.30)	0.298	1.03 (0.87–1.22)	0.752
Chronic kidney disease	0.83 (0.25–2.74)	0.371	2.45 (0.91–6.59)	0.150
Diabetes mellitus	1.89 (0.62–5.79)	0.282	1.91 (0.74–4.92)	0.226
Indwelling urinary catheters	2.11 (0.37–12.05)	< 0.001*	5.43 (1.39–21.23)	< 0.001*
No infectious disease team consult	6.38 (1.96–20.72)	< 0.001*	2.90 (1.04–8.11)	0.032*
Previous exposure to antibiotics	2.87 (0.64–12.79)	0.351	2.04 (0.67–6.27)	0.168
Non-effective empirical therapy	1.46 (0.35–5.62)	0.824	1.90 (0.52–6.89)	0.828
Hospital-acquired MRSA	7.05 (1.61–30.85)	0.021*	6.12 (1.81–20.72)	0.017*
Healthcare-associated MRSA	3.03 (0.95–9.64)	0.463	3.19 (1.30–7.81)	0.043*
Hypoalbuminemia (< 29 g/L)	1.66 (0.51–5.42)	0.386	3.31 (1.25–8.79)	0.017*
Polymicrobial bacteremia	3.35 (0.43–1.07)	0.032*	2.50 (0.95–6.58)	0.282
Inappropriate targeted treatment	3.38 (0.54–21.19)	0.694	8.08 (1.15–56.86)	0.024*
Inappropriate treatment duration	1.49 (0.48–4.65)	0.160	1.62 (0.61–4.32)	0.410

*p-value < 0.05 indicates statistical significance

**Fig. 1:**
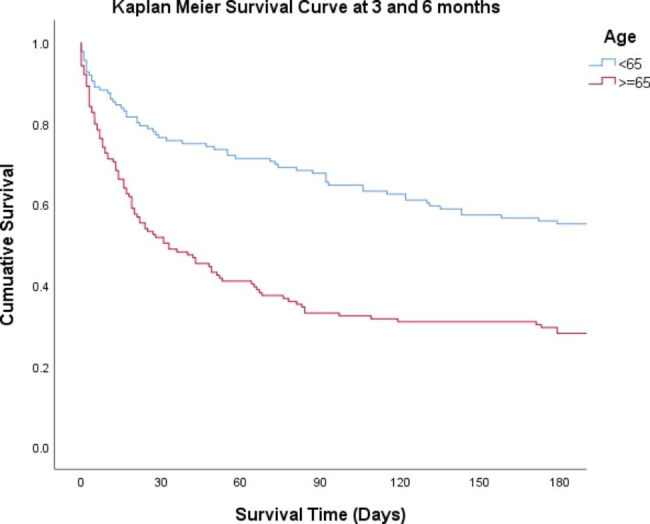
Kaplan-Meier survival curve for mortality. Time to death in days for individuals aged 18–64 years (blue) and 65 years and older (green) with MRSA bacteremia

The overall survival rates were significantly lower among older patients (p ≤ 0.002) when measured across three different time points (14, 30, and 180 days) (Table 5). The Kaplan-Meier curve generated from the survival analysis using the log-rank (Mantel-Cox) test showed a significantly lower survival among older patients (p = 0.025) (Fig. 1).

**Table 5:** Patient survival by age groups

Age	14-Day Survival, mean (95% CI)	30-Day Survival, mean (95% CI)	180-Day Survival, mean (95% CI)
18–64 years	12.54 (11.91–13.18)	25.40 (23.81–26.99)	122.69 (110.46-134.92)
≥ 65	11.05 (10.24–11.86)	20.27 (18.34–22.19)	73.05 (60.55–85.55)
p-value (Log-rank test)	0.002*	< 0.001*	< 0.001*

*p-value < 0.05 indicates statistical significance

## Discussion

In this study, MRSA bacteremia was mainly acquired in the hospital (52.7%) and other healthcare facilities (35.6%). Similar observations were noted in other countries in Southeast Asia [26, 27]. Our findings were consistent with previous reports of HA and HCA infections being significant risk factors for mortality in patients with MRSA bacteremia [28, 29]. Although HCA-MRSA is commonly associated with long-term care facilities (LTCF) or nursing homes [30, 31], most of the HCA-MRSA cases in our study population were from a recent hospitalization. However, it is important to note that among the recently hospitalized patients, the information on whether the patients were residing in LCTF was not captured in this study.

Infections in older persons accounted for over half of the cases of MRSA bacteremia identified in this study. Previously conducted epidemiological studies showed that S. *aureus* bacteremia is higher in the older population [32, 33]. Our data showed that the risk for 30-day mortality was three times higher in older persons (AOR 3.36, 95% CI 1.24–9.13, p = 0.024), consistent with previous studies that reported age as the significant risk factor for short- and long-term mortality in patients with MRSA bacteremia [29, 32]. Multiple factors including epidemiological conditions, concurrent medical illness, increased use of invasive devices, longer hospital stay, malnutrition, as well as physiologic and anatomical age-dependent changes, such as immune-senescence, likely attributed to the increased vulnerability of older persons to infections and leading to significantly higher mortality [32, 34–38].

The presence of an indwelling urinary catheter was significantly associated with mortality in patients with MRSA bacteremia in this study. Indwelling urinary catheterization poses serious complications and increases the risk of bloodstream infection in older patients [33, 39]. Previous studies have shown urinary catheters to be an independent risk factor for HA-MRSA [40–42]. Previously, the association between urinary catheterization and mortality due to MRSA bacteremia has been mostly inconclusive [43, 44]. However, our data showed a positive correlation between indwelling urinary catheters and mortality due to MRSA. Although *S. aureus* is not typically considered a major cause of UTI, recent epidemiological studies indicate that *S. aureus* is an emerging cause of UTI in specific patient populations, such as pregnant women and those with complicated UTI [45]. Previous studies have shown that the placement of a catheter into the bladder leads to a specific and localized inflammatory response resulting in the release of the host protein fibrinogen, which accumulates in the bladder and on the catheter [46]. This shows that urinary catheterization alters the urinary tract environment and that MRSA may exploit the presence of this host protein to cause disseminated disease [46].

We observed that the rate of CLABSI was significantly higher in younger patients in this cohort. This is likely due to the higher number of younger patients (26.6%) having central venous catheters (CVC) compared to older persons (18%). The high rates of CLABSI in both groups may be attributable to the suboptimal compliance to central line care bundles leading to an increased introduction of infection during insertion and manipulation of the lines [47]. In this study, older patients were more prone to skin and soft tissue infections. The older population is more susceptible to skin wounds due to changes associated with the aging process that increase skin fragility [48]. The rates of primary bacteremia are known to be higher in older patients (aged > 75 years) [33]. However, this study observed no significant difference in primary bacteremia between the older and younger patients.

Consistent with previous reports, the Charlson Comorbidity Index and Pitt bacteremia scores were observed to be higher in older patients [44, 49]. Antibiotic exposure not only increases the risk of MRSA infection or colonization by approximately two folds, but it is also one of the reasons for the increase in MRSA rates in the community [50, 51]. In the present study, more than half of the younger (64.7%) and older (60.4%) patients had exposure to antibiotics in the last 3 months, which might be attributable to the MRSA bacteremia in these patients.

Previous studies have highlighted the relationship between inappropriate empirical therapy with increased mortality [52, 53]. The present study showed that 14.2% and 18.3% of the younger and older patients received effective empirical antibiotics. Ineffective empirical therapy was, however, not found to be a significant risk for mortality in our study. The present study revealed 22.6% of the older patients did not receive appropriate targeted antibiotics and 56.6% received inappropriate treatment duration. Multivariate analysis showed that there were 8.08 times higher odds for 30-day mortality in those who received inappropriate targeted therapy. The reasons for this may be due to poor knowledge of the significance of a positive culture and management of MRSA bacteremia [54]. Furthermore, the medical teams may have taken a conservative or palliative approach in the management of frail, older patients leading to the decision not to subject the patient to vancomycin, an antibiotic that has been associated with significant nephrotoxicity [55].

In the present study, approximately one-third of the older patients received ID consultations. The mortality risks on days 14 and 30 were six and three times higher in patients who did not get an ID team input compared to those who did. Similarly, studies by Buehrle et al. [56] and Pragman et al. [57] showed that patients who had an ID consult were more likely to receive guidelines-congruent management and had significantly better outcomes. It is now accepted that a low albumin level is associated with higher mortality in patients with sepsis [58]. This is due to a dysregulation in albumin gene expression caused by elevated cytokine levels in septic conditions [59]. Similarly, our study found a significant association between hypoalbuminemia and 30-day mortality in patients with MRSA bacteremia.

There are several limitations to our study. Firstly, this is a single-center study hence the findings might not represent the entire population and the retrospective nature of this study is subjected to the potential effects of unmeasured variables, missing information, selection and recall biases [60]. Secondly, there were cases where the patients did not receive appropriate antimicrobials and treatment duration (died within 48 h of positive culture), which might consequently impact the mortality rates. Finally, as crude mortality was recorded in this study, the cause of death could be due to underlying co-morbidities instead of the infection. Nonetheless, our study adds to the literature on MRSA bacteremia in the older population, which is largely overlooked in this region [11, 61]. The results of this study highlight the importance of developing and implementing hospital protocols designed to improve infection control practices in older patients such as care of vascular devices, urinary catheters, and pressure ulcers as well as antimicrobial stewardship programs such as MRSA bacteremia clinical pathway, to improve patient outcomes [62]. The risk factors identified in the present study can be used to develop and validate a scoring system for MRSA bacteremia-associated mortality.

## Conclusions

The incidence of MRSA bacteremia in healthcare settings is higher than in the community. Older age, higher Pitt bacteremia scores, HA-MRSA and HCA-MRSA, hypoalbuminemia, polymicrobial infection, presence of indwelling urinary catheters, lack of ID team consultation, and inappropriate targeted treatment were the significant risk factors for mortality in MRSA bacteremia. Older patients had a three times higher mortality risk than younger patients. Specific policies and guidelines for the prevention and management of MRSA bacteremia are urgently needed to address the rising rates of infection as well as to improve outcomes, particularly in older patients.

## Data Availability

All data generated or analyzed during this study are included in this published article.

## List of Abbreviations

AOR: Adjusted odds ratios
CA-MRSA: Community-acquired methicillin-resistant *Staphylococcus aureus*
CA-UTI: Catheter-associated urinary tract infection
CDC: Centre for Disease Control and Prevention
CI: Confidence intervals
CLABSI: Central line-associated bloodstream infection
CLSI: Clinical and Laboratory Standards Institute
HA-MRSA: Hospital-acquired methicillin-resistant *Staphylococcus aureus*
HCA-MRSA: Healthcare-associated methicillin-resistant *Staphylococcus aureus*
ICU: Intensive care unit
MRSA: Methicillin-resistant *Staphylococcus aureus*
MSSA: Methicillin-susceptible *Staphylococcus aureus*
SD: Standard deviation
SSI: Surgical site infection
UMMC: University Malaya Medical Centre

## References

[1] United Nations, Department of Economic and Social Affairs, Population Division. : World Population Prospects 2019. https://population.un.org (2019). Accessed 8 Jul 2022.

[2] Department of Statistics Malaysia: Current population estimates, Malaysia., 2022. https://www.dosm.gov.my/v1/index.php?r=column/cthemeByCat&cat=155&bul_id=dTZXanV6UUdyUEQ0SHNWOVhpSXNMUT09&menu_id=L0pheU43NWJwRWVSZklWdzQ4TlhUUT09 (2022). Accessed 9 Jan 2023.

[3] Esme M, Topeli A, Yavuz BB, Akova M (2019). Infections in the elderly critically-ill patients. Front Med.

[4] Pomorska-Wesołowska M, Różańska A, Natkaniec J, Gryglewska B, Szczypta A, Dzikowska M (2017). Longevity and gender as the risk factors of methicillin-resistant *Staphylococcus aureus* infections in southern Poland. BMC Geriatr.

[5] Taylor TA, Unakal CG. *Staphylococcus aureus*. StatPearls, Treasure Island. 2018. https://www.ncbi.nlm.nih.gov/books/NBK441868/. Accessed 15 Jun 2022.

[6] Goering RV, Dockrell HM, Zuckerman M, Chiodini PL. Infections of the skin, soft tissue, muscle and associated systems. In: Mims’ Medical Microbiology and Immunology (6th Edition). Elsevier Ltd. 2019. https://www.clinicalkey.com/#!/content/book/3-s2.0-B9780702071546000278. Accessed 9 Jan 2023.

[7] Thomer L, Schneewind O, Missiakas D (2016). Pathogenesis of *Staphylococcus aureus* bloodstream infections. Annu Rev Pathol.

[8] Lakhundi S, Zhang K (2018). Methicillin-resistant *Staphylococcus aureus*: molecular characterization, evolution, and epidemiology. Clin Microbiol Rev.

[9] World Health Organization (WHO). Antimicrobial resistance: Global report on surveillance. World Health Organization, Geneva, Switzerland. 2014. https://www.who.int/antimicrobial-resistance/publications/surveillancereport/en/. Accessed 8 Jul 2022.

[10] Murray CJL, Ikuta KS, Sharara F, Swetschinski L, Robles Aguilar G, Gray A (2022). Global burden of bacterial antimicrobial resistance in 2019: a systematic analysis. Lancet.

[11] Chen CJ, Huang YC (2014). New epidemiology of *Staphylococcus aureus* infection in Asia. Clin Microbiol Infect.

[12] Boswihi SS, Udo EE (2018). Methicillin-resistant *Staphylococcus aureus*: an update on the epidemiology, treatment options and infection control. Curr Med Res Pract.

[13] Hassoun A, Linden PK, Friedman B (2017). Incidence, prevalence, and management of MRSA bacteremia across patient populations – a review of recent developments in MRSA management and treatment. Crit Care.

[14] Charlson ME, Pompei P, Ales KL, MacKenzie CR (1987). A new method of classifying prognostic comorbidity in longitudinal studies: development and validation. J Chronic Dis.

[15] Magiorakos AP, Srinivasan A, Carey RB, Carmeli Y, Falagas ME, Giske CG (2012). Multidrug-resistant, extensively drug-resistant and pandrug-resistant bacteria: an international expert proposal for interim standard definitions for acquired resistance. Clin Microbiol Infect Dis.

[16] Friedman ND, Kaye KS, Stout JE, McGarry SA, Trivette SL, Briggs JP (2002). Health care-associated bloodstream infections in adults: a reason to change the accepted definition of community-acquired infections. Ann Intern Med.

[17] Lenz R, Leal JR, Church DL, Gregson DB, Ross T, Laupland KB (2012). The distinct category of healthcare associated bloodstream infections. BMC Infect Dis.

[18] Rodríguez-Baño J, López-Prieto MD, Portillo MM, Retamar P, Natera C, Nuño E (2010). Epidemiology and clinical features of community-acquired, healthcare-associated and nosocomial bloodstream infections in tertiary-care and community hospitals. Clin Microbiol Infect.

[19] Horan TC, Andrus M, Dudeck MA (2008). CDC/NHSN surveillance definition of health care-associated infection and criteria for specific types of infections in the acute care setting. Am J Infect Control.

[20] Stevens DL, Bisno AL, Chambers HF, Dellinger EP, Goldstein EJ, Gorbach SL (2014). Practice guidelines for the diagnosis and management of skin and soft tissue infections: 2014 update by the infectious Diseases Society of America. Clin Infect Dis.

[21] Clinical and Laboratory Standards Institute (CLSI) (2011). Performance standards for antimicrobial susceptibility testing: twenty-first informational supplement.

[22] Liu C, Bayer A, Cosgrove SE, Daum RS, Fridkin SK, Gorwitz RJ (2011). Clinical practice guidelines by the infectious Diseases Society of America for the treatment of methicillin-resistant *Staphylococcus aureus* infections in adults and children. Clin Infect Dis.

[23] Hawkins C, Huang J, Jin N, Noskin GA, Zembower TR, Bolon M (2007). Persistent *Staphylococcus aureus* bacteremia: an analysis of risk factors and outcomes. Arch Intern Med.

[24] Welsh KJ, Skrobarcek KA, Abbott AN, Lewis CT, Kruzel MC, Lewis EM (2011). Predictors of relapse of methicillin-resistant *Staphylococcus aureus* bacteremia after treatment with vancomycin. J Clin Microbiol.

[25] de Kraker MEA, Wolkewitz M, Davey PG, Grundmann H (2011). Clinical impact of antimicrobial resistance in european hospitals: excess mortality and length of hospital stay related to methicillin-resistant *Staphylococcus aureus* bloodstream infections. Antimicrob Agents Chemother.

[26] Jaganath D, Jorakate P, Makprasert S, Sangwichian O, Akarachotpong T, Thamthitiwat S (2018). *Staphylococcus aureus* bacteremia incidence and methicillin resistance in rural Thailand, 2006–2014. Am J Trop Med.

[27] Lim WW, Wu P, Bond HS, Wong JY, Ni K, Seto WH (2019). Determinants of methicillin-resistant *Staphylococcus aureus* (MRSA) prevalence in the Asia-Pacific region: a systematic review and meta-analysis. J Glob Antimicrob Resist.

[28] Aratani T, Tsukamoto H, Higashi T, Kodawara T, Yano R, Hida Y (2021). Association of methicillin resistance with mortality of hospital-acquired *Staphylococcus aureus* bacteremia. J Intern Med Res.

[29] Ayau P, Bardossy AC, Sanchez G, Ortiz R, Moreno D, Hartman P (2017). Risk factors for 30-day mortality in patients with methicillin-resistant *Staphylococcus aureus* bloodstream infections. Int J Infect Dis.

[30] Faria NA, Oliveira DC, Westh H, Monnet DL, Larsen AR, Skov R (2005). Epidemiology of emerging methicillin-resistant *Staphylococcus aureus* (MRSA) in Denmark: a nationwide study in a country with low prevalence of MRSA infection. J Clin Microbiol.

[31] Roghmann M-C, Johnson JK, Sorkin JD, Langenberg P, Lydecker A, Sorace B (2015). Transmission of methicillin-resistant *Staphylococcus aureus* (MRSA) to healthcare worker gowns and gloves during care of nursing home residents. Infect Control Hosp Epidemiol.

[32] Guillamet MCV, Vazquez R, Deaton B, Shroba J, Vazquez L, Mercier R-C (2018). Host-pathogen-treatment triad: host factors matter most in methicillin-resistant *Staphylococcus aureus* bacteremia outcomes. Antimicrob Agents Chemother.

[33] Yahav D, Eliakim-Raz N, Leibovici L, Paul M (2016). Bloodstream infections in older patients. Virulence.

[34] Mejer N, Westh H, Schønheyder HC, Jensen AG, Larsen AR, Skov R (2012). Stable incidence and continued improvement in short-term mortality of *Staphylococcus aureus* bacteraemia between 1995 and 2008. BMC Infect Dis.

[35] Andreassen AES, Jacobsen CM, de Blasio B, White R, Kristiansen IS, Elstrøm P (2017). The impact of methicillin-resistant *S. aureus* on length of stay, readmissions and costs: a register-based case-control study of patients hospitalized in Norway. Antimicrob Resist Infect Control.

[36] Shuping LL, Kuonza L, Musekiwa A, Iyaloo S, Perovic O (2017). Hospital-associated methicillin-resistant *Staphylococcus aureus*: a cross-sectional analysis of risk factors in south african tertiary public hospitals. PLoS ONE.

[37] Gavazzi G, Krause K-H (2002). Ageing and infection. Lancet Infect Dis.

[38] Leibovici-Weissman Y, Tau N, Yahav D (2021). Bloodstream infections in the elderly: what is the real goal?. Aging Clin Exp Res.

[39] Carnicer-Pont D, Bailey KA, Mason BW, Walker AM, Evans MR, Salmon RL (2006). Risk factors for hospital-acquired methicillin-resistant *Staphylococcus aureus* bacteraemia: a case-control study. Epidemiol Infect.

[40] Pogorzelska-Maziarz M, Furuya EY, Larson EL (2013). Risk factors for methicillin-resistant *Staphylococcus aureus* bacteraemia differ depending on the control group chosen. Epidemiol Infect.

[41] Porto JP, Santos RO, Gontijo Filho PP, Ribas RM (2013). Active surveillance to determine the impact of methicillin resistance on mortality in patients with bacteremia and influences of the use of antibiotics on the development of MRSA infection. Rev Soc Bras Med Trop.

[42] Selvey LA, Whitby M, Johnson B (2000). Nosocomial methicillin-resistant *Staphylococcus aureus* bacteremia: is it any worse than nosocomial methicillin-sensitive *Staphylococcus aureus* bacteremia?. Infect Control Hosp Epidemiol.

[43] Bakowski E, Wey SB, Medeiros EAS (2008). Risk factors for bacteraemia and predictors of mortality of patients with bloodstream infection with methicillin-resistant *Staphylococcus aureus*. Am J Infect Dis.

[44] Cuervo G, Gasch O, Shaw E, Camoez M, Domínguez M, Padilla B (2016). Clinical characteristics, treatment and outcomes of MRSA bacteraemia in the elderly. J Infect.

[45] Walker JN, Flores-Mireles AL, Pinkner CL, Schreiber HL, Joens MS, Park AM (2017). Catheterization alters bladder ecology to potentiate *Staphylococcus aureus* infection of the urinary tract. Proc Natl Acad Sci U S A.

[46] Flores-Mireles AL, Pinkner JS, Caparon MG, Hultgren SJ (2014). EbpA vaccine antibodies block binding of *Enterococcus faecalis* to fibrinogen to prevent catheter-associated bladder infection in mice. Sci Transl Med.

[47] Lee KH, Cho NH, Jeong SJ, Kim MN, Han SH, Song YG (2018). Effect of central line bundle compliance on central line-associated bloodstream infections. Yonsei Med J.

[48] Farage MA, Miller KW, Elsner P, Maibach HI (2012). Characteristics of the aging skin. Adv Wound Care.

[49] Marchaim D, Kaye KS, Fowler VG, Anderson DJ, Chawla V, Golan Y (2010). Case-control study to identify factors associated with mortality among patients with methicillin-resistant *Staphylococcus aureus* bacteraemia. Clin Microbiol Infect.

[50] Khan A, Wilson B, Gould IM (2018). Current and future treatment options for community-associated MRSA infection. Expert Opin Pharmacother.

[51] Tacconelli E, De Angelis G, Cataldo MA, Pozzi E, Cauda R (2007). Does antibiotic exposure increase the risk of methicillin-resistant *Staphylococcus aureus* (MRSA) isolation? A systematic review and meta-analysis. J Antimicrob Chemother.

[52] Gradel KO, Jensen US, Schønheyder HC, Østergaard C, Knudsen JD, Wehberg S (2017). Impact of appropriate empirical antibiotic treatment on recurrence and mortality in patients with bacteraemia: a population-based cohort study. BMC Infect Dis.

[53] Yoon YK, Park DW, Sohn JW, Kim HY, Kim Y-S, Lee C-S (2016). Effects of inappropriate empirical antibiotic therapy on mortality in patients with healthcare-associated methicillin-resistant *Staphylococcus aureus* bacteremia: a propensity-matched analysis. BMC Infect Dis.

[54] Lambert M (2011). IDSA guidelines on the treatment of MRSA infections in adults and children. Am Fam Physician.

[55] Filippone EJ, Kraft WK, Farber JL (2017). The nephrotoxicity of vancomycin. Clin Pharmacol Ther.

[56] Buehrle K, Pisano J, Han Z, Pettit NN (2017). Guideline compliance and clinical outcomes among patients with *Staphylococcus aureus* bacteremia with infectious diseases consultation in addition to antimicrobial stewardship-directed review. Am J Infect Control.

[57] Pragman AA, Kuskowski MA, Abraham JM, Filice GA (2012). Infectious disease consultation for *Staphylococcus aureus* bacteremia improves patient management and outcomes. Infect Dis Clin Pract (Baltim Md).

[58] Takegawa R, Kabata D, Shimizu K, Hisano S, Ogura H, Shintani A (2019). Serum albumin as a risk factor for death in patients with prolonged sepsis: an observational study. J Crit Care.

[59] Yin M, Si L, Qin W, Li C, Zhang J, Yang H (2018). Predictive value of serum albumin level for the prognosis of severe sepsis without exogenous human albumin administration: a prospective cohort study. J Intensive Care Med.

[60] Talari K, Goyal M (2020). Retrospective studies - utility and caveats. J R Coll Physicians Edinb.

[61] You JHS, Choi K-W, Wong T-Y, Ip M, Ming W-K, Wong RY-K (2017). Disease burden, characteristics, and outcomes of methicillin-resistant *Staphylococcus aureus* bloodstream infection in Hong Kong. Asia Pac J Public Health.

[62] Duerden B, Fry C, Johnson AP, Wilcox MH (2015). The control of methicillin-resistant *Staphylococcus aureus* bloodstream infections in England. Open Forum Infect Dis.

